# Long-Term Health Impacts of Wildfire Exposure: A Retrospective Study Exploring Hospitalization Dynamics Following the 2016 Wave of Fires in Israel

**DOI:** 10.3390/ijerph19095012

**Published:** 2022-04-20

**Authors:** Odeya Cohen, Stav Shapira, Eyal Furman

**Affiliations:** 1 Department of Nursing, Recanati School for Community Health Professions, Faculty of Health Sciences, Ben-Gurion University of the Negev, P.O. Box 653, Beer Sheva 8410501, Israel; 2 School of Public Health, Faculty of Health Sciences, Ben-Gurion University of the Negev, P.O. Box 653, Beer Sheva 8410501, Israel; stavshap@bgu.ac.il; 3 Maccabi Healthcare Services, Haifa 3508510, Israel; furman_e@mac.org.il

**Keywords:** climate change, natural disasters, wildfires, long-term health impact, socio-economic factors, healthcare utilization

## Abstract

Background: Climate-related events, including wildfires, which adversely affect human health, are gaining the growing attention of public-health officials and researchers. Israel has experienced several disastrous fires, including the wave of fires in November 2016 that led to the evacuation of 75,000 people. The fires lasted six days (22–27 November) with no loss of life or significant immediate health impacts. The objective of this study is to explore the long-term hospitalization dynamics in a population exposed to this large-scale fire, including the effects of underlying morbidity and socio-economic status (SES). Methods: This is a retrospective crossover study, conducted in 2020, analyzing the electronic medical records of residents from areas exposed to a wildfire in northern Israel. The study spans from one year before exposure to two years after it (22 November 2015–27 November 2018). The hospitalization days during the study period were analyzed using the Poisson regression model. The rate of hospitalization days along with 95% confidence intervals (CIs) were plotted. Results: The study included 106,595 participants. The median age was 37 (IQR = 17–56), with a mean socio-economic ranking of 6.47 out of 10 (SD = 2.01). Analysis revealed that people with underlying morbidity were at greater risk of experiencing long-term effects following fires, which was manifested in higher hospitalization rates that remained elevated for two years post-exposure. This was also evident among individuals of low socio-economic status without these background illnesses. Conclusions: Healthcare services should prepare for increased hospitalization rates during the two years following wildfires for populations with underlying morbidity and those of low socio-economic status. Implementing preventive-medicine approaches may increase the resiliency of communities in the face of extreme climate-related events and prevent future health burdens. Additional research should focus on the specific mechanisms underpinning the long-term effects of wildfire exposure.

## 1. Introduction

Wildfires are known to adversely affect human health through a variety of mechanisms and are thus gaining attention as a major public health concern [1,2,3,4]. Their main—and most studied—influence on human health is exposure to smoke containing elevated ambient air pollutants [5]. Despite some inconsistencies, epidemiological evidence has broadly associated smoke exposure with respiratory and cardiovascular morbidity, all-cause mortality [6]; ophthalmic effects such as eye irritation [7]; and adverse pregnancy and birth outcomes such as increased preterm deliveries, low birth weight, and stillbirths [8,9]. Other reported mechanisms that lead to harmful health effects are related to direct flame and heat exposure causing burns [10]. Water and land pollution resulting from the incineration of various materials may also lead to toxic chemical exposure [11,12]. Decreased access to healthcare services may also occur due to traffic congestion caused by population evacuation [13], or the need to evacuate healthcare institutions directly impacted by the fire [14], potentially leading to delays in receiving medical aid or disruptions to the continuity of routine care. Population evacuation may also lead to difficulty accessing vital resources such as food and water [15]—posing a significant threat to vulnerable populations such as young children and those suffering from chronic illnesses requiring special nutrition, such as diabetics.

Most studies concerning the health effects associated with exposure to wildfires have focused on short-term outcomes, with relatively sparse evidence of long-term health consequences. A recent review that evaluated the long-term health impacts and health needs among populations exposed to wildfires reported an increased risk of premature deaths, respiratory complications, and population-based increases in cancer risk [16]. However, the researchers stressed that the existing evidence is scant and pointed to significant gaps in the literature concerning the demographic profile of vulnerable populations such as medically vulnerable and socially disadvantaged populations, despite evidence that these populations are more susceptible to the adverse effects of wildfires [1,2,6]. Another recent Canadian study examined the effects of an extreme wildfire on the long-term mental health of the population that was evacuated. The findings indicated relatively increased rates of major depressive disorder, anxiety, and post-traumatic stress disorder eighteen months following exposure. Limited or non-existent social and municipal support after the wildfire was associated with an increased likelihood of experiencing adverse mental impacts [17].

Climate change has resulted in prolonged and more frequent heatwaves, increasing the frequency and intensity of wildfires [18]. Israel has experienced several disastrous and deadly fires including the 2010 mega-fire on Mount Carmel, and the wave of fires in November 2016—the focus of the present study. The 2016 wave of fires lasted six days (22–27 November) and in terms of property and environmental damage, this wave of fires is considered the worst in the history of Israel. Over 1700 fires were reported in various locations across the country. More than 10,000 acres were burned, and approximately 2000 residential structures were damaged. Of these, approximately 600 were destroyed completely. The largest and most destructive fires spread across the city of Haifa, the third-largest city in Israel with a population of 280,000 residents. The spread of the fires led to the evacuation of 68,000 people, almost 25% of the urban population in the Haifa Bay region [19]. Despite extensive damage, no loss of life or significant direct health impacts were reported.

As projections indicate the Mediterranean region to become dryer and warmer, resulting in increased fire risk [20], it is important to study the health effects of wildfires and identify those populations most susceptible to such events. Based on former studies, hospitalization rates are a common indicator for evaluating the impact of wildfires on human health, with clinical and logistical implications for preparedness [5,6,21]. This study aims to explore long-term hospitalization dynamics in a population that was exposed to a large-scale fire, including the effects of underlying morbidity and socio-economic status on hospitalizations.

## 2. Materials and Methods

### 2.1. Design and Setting of the Study—A Retrospective Study

#### 2.1.1. Data Collection

Maccabi Health Services (MHS) is Israel’s second-largest health fund, providing medical services to 2.3 million members, about one-quarter of the Israeli population. In the northern district of MHS, there are 430,000 members with socio-demographic characteristics similar to the general population in terms of age, gender, ethnicity, and socio-economic status. Healthcare in Israel is primarily provided at the community level, by a large network of community-based clinics [22]. A primary clinic is assigned to each MHS member, usually based on geographical proximity to the member’s home. For this project, we relied on MHS data retrieved in January 2020 to obtain information on 106,595 members whose primary clinic was in the area impacted by the 2016 wildfires in the Haifa Bay area. As detailed in the previous section, the entire city of Haifa was highly impacted by the wildfires. Several combustion events occurred within the city itself, wreaking havoc, producing heavy smoke in several neighborhoods, and leading to massive population evacuation. Thus, our inclusion criteria were: (1) residents of areas that had been evacuated in the fire of 2016; (2) belonging to MHS clinics in those areas. We used a continuous sampling method in which a potential participant who met the general inclusion criteria entered the study without further constraints.

For each study participant, we obtained information on:

(A) Morbidity factors based on established MHS registries—We used MHS registries for four chronic morbidities for each patient: cardiovascular, obstructive pulmonary disease, overweight, and diabetes. We chose to focus on these specific morbidities following the well-documented short-term impacts of wildfires on them [4,6,7]. Overweight was chosen due to its increased prevalence in the general population and the association of obesity with other non-communicable diseases. The registries are updated automatically every day, drawing data from many sources: diagnoses, hospital discharge codes, billing information from providers, and prescription information [23]. The study population was divided into two sub-populations: the offset population that did not appear in any of these registries at the time of exposure (*n* = 56,966, 54%), and those with one or more of the chronic morbidities described above at the time of exposure (*n* = 49,629, 46%). In this study, we did not measure co-morbidity because our pre-analysis to examine the impact of each chronic condition on the hospitalization rate during the study periods revealed similar findings for each.
(B) Hospitalization over a three-year period—We included the hospitalization days (based on the number of overnight stays) from all types of Israeli hospitals in all wards, except the maternity wards. The hospitalization information spanned from one year pre-exposure (22 November 2015) to two years post-exposure (27 November 2018).
(C) Personal characteristics (age and gender).
(D) Socio-economic status (SES) on a scale of 1–10 (1 = low to 10 = high), based on a poverty index calculated for each residential location. The enumeration area was calculated for each location based on a geographical unit (usually consisting of several thousand individuals) defined by the Israeli Central Bureau of Statistics, based on the homogeneity of the socio-demographic characteristics of the residing population. The poverty index is based on several factors, including: educational level, physical conditions, household income, crowding, and car ownership [24]. The study was approved by MHS’s Institutional Review Board for the Protection of Human Subjects (0028-19-MHS).

#### 2.1.2. Statistical Analysis

Descriptive statistics were used to characterize the study population. SES was divided into three sub-populations: low (1–4), medium (5–7), and high (8–10). The hospitalization dynamic over the study periods was analyzed through three methods: (1) The means of hospitalization days over study periods. (2) A generalized linear model with family set to Negative Binomial, and log link was used as an approximation to Poisson regression with zero inflation. The dependent variable was the number of hospitalization days during the pre-exposure year, the year after the exposure, and two years after the exposure. The independent variables included: age; gender; time-varying indicators for the three time periods; SES categories, and an indicator variable for individuals who appeared in at least one of the morbidity registries mentioned above, with second-order interactions between time periods, SES categories, and morbidity. (3) The rates of hospitalization days along with 95% confidence intervals (CIs) were plotted based on the estimated means of hospitalization days from the regression model.

## 3. Results

The study included 106,595 participants, of whom 51.8% were women (*n* = 55,291), with a median age (in 2016) of 37 (IQR = 17–56). Table 1 describes the socio-demographic characteristics of the study population. The mean amount of hospitalization days over each of the study periods was: (a) during the pre-exposure year: 0.15 (*SD* = 0.67) days; (b) during the first year post-exposure: 0.17 (*SD* = 0.75) days; and (c) through the second year post-exposure: 0.15 (*SD* = 0.70) days.

About 46% (*n* = 49,629) of the participants appeared in one registry or more. Table 2 presents the socio-demographic characteristics of the participants in each registry. Participants with obstructive pulmonary disease had a higher median age of 70 (IQR = 64–77) and the lowest SES 6.19 (*SD* = 2.21, 2–10). Overweight participants had a lower median age = 50 (IQR = 36–62). Fewer than 50% of the participants with cardiovascular diseases were women (40.6%). The percentage of participants born in Israel was lower in all registries than their ratio in the study population.

The results of the Poisson regression model are presented in Table 3 (Likelihood Ratio Chi-Square = 28,781.334, *df* = 19, *p* < 0.001). Among the main effects, age at the time of exposure and gender (male vs. female) were risk factors. In regard to study periods, the two years post-exposure show a significant risk compared to the pre-exposure year. Among main effects and interactions, underlying morbidity presented the highest risk (exp(B) = 1.593, 95% CI 1.466–1.730). High SES was found as a protective factor compared to low SES (exp(B) = 0.678, 95% CI 0.621–0.741).

**Figure 1:**
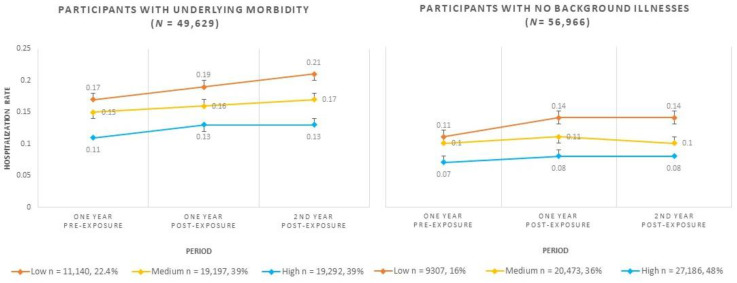
Hospitalization rates by SES and morbidity status based on estimated means of Poisson regression during the study period (2015–2018).

Based on the estimated means of the regression model, hospitalization patterns both pre- and post-exposure revealed that participants with underlying morbidity show an increase in hospitalization rates that persists two years post-exposure. Furthermore, the disparity in hospitalization rates between the low and high SES groups increased from 54% pre-exposure to 61% at two years post-exposure. Among participants with no underlying morbidity, only those of low SES demonstrated a significant increase in hospitalization rates post-exposure, from a disparity of 57% pre-exposure to 75% at two years post-exposure. Figure 1 and Table A1 present the hospitalization rates (along with 95% CIs) during study periods according to sub-population SES.

## 4. Discussion

This study was designed to explore the long-term effects of exposure to a large-scale fire, comparing pre- and post-exposure hospitalization dynamics. The results indicate that individuals with underlying morbidity and those with low SES are at increased risk for experiencing long-term health effects, which manifested in higher hospitalization rates that remained elevated for two years post-exposure. Another important finding relates to the growing gap in hospitalization rates between the low and high SES groups.

These findings demonstrate the compounding long-term effects on both health and healthcare utilization following wildfires. Furthermore, the current results stress that structural conditions of disadvantage (i.e., low SES) undermine the recovery capacities of populations exposed to natural disasters such as wildfires [25]. The results resonate with previous studies which indicated that medically vulnerable and socially disadvantaged populations are susceptible to the immediate impacts of wildfires including health consequences [5,6], property damage, and other economic impacts such as loss of livelihood [26]. With regard to the long-term effects of wildfire exposure, there is well-known difficulty in determining causality as well as in identifying the specific mechanisms and pathways linking exposure and outcome [27]. In the current context, one can speculate that additional personal or environmental factors—that may have changed over the study period and were not controlled in the current analysis—have also contributed to the long-term changes in hospitalization dynamics observed. A particular example of such a factor is the well-documented exposure to air pollutants from the petrochemical industry located in the Haifa Bay [28]. However, the present study clearly indicates that the contribution of the initial exposure, especially when combined with specific pre-existing risk factors, should not be overlooked, and further raises important questions regarding the specific mechanisms underpinning the observed changes. An additional limitation of this study is related to the nature of the data and the use of the participants’ primary clinic address as a proxy for personal exposure to the wildfires. This method does not allow for a clear verification of the participants’ presence in the Haifa Bay during the event and may lead to a potential bias. However, our study relates to ‘exposure’ in this specific context in a broad sense—i.e., even if a person was not present during the event itself, he/she was probably indirectly impacted, for example, through experiencing property damage, or even by witnessing the destruction caused to their residential environment.

Thus, a possible path to long-term health deterioration following wildfires may stem from the psychological effects of these devastating events. A recent review pointed to the far-reaching mental health effects of wildfire exposure, revealing elevated rates of various conditions such as anxiety, depression, and insomnia between 6 and 18 months following a massive wildfire in Canada [29]. These findings were supported by two other Israeli studies. One study found increased mental distress among firefighters who responded to the 2010 Mount Carmel fire during the three years following the fire [30]. Another study reported elevated levels of distress among community-dwelling individuals affected by the fire explored in this current study four months following the fire [31]. Ample evidence identifies the strong link between mental disorders and physical health [32]; this association is even stronger among individuals with chronic health conditions such as COPD [33], diabetes [34], and cardiovascular diseases [35]. Thus, it is possible that mental health effects following exposure to the 2016 wave of fires played a crucial role in the long-term adverse health effects that were shown in the current study. This remains an issue for further exploration.

In a broader context, as the frequency and intensity of climate-related extreme events are expected to increase, it is of the utmost importance to invest time and resources into mitigating their adverse health effects [2]. One course of action to mitigate the long-term outcomes of wildfire exposure would be to improve the delivery of preventive services in the primary-care setting provided in the community. This would reduce morbidity in the pre-exposure phase, especially among vulnerable populations such as socio-economically deprived individuals, and potentially have an effect on the health outcomes of disadvantaged populations both on a daily basis and, as suggested by our results, following emergencies. Additionally, future studies should focus on the mechanisms underpinning long-term health effects following wildfire exposure. Gaining such understanding will advance current knowledge regarding determinants of disaster vulnerability and health risks.

## 5. Conclusions

Despite the limitations mentioned above, the current study was exploratory and provided new evidence of the long-term adverse health consequences of wildfire exposure. The World Health Organization (WHO) called for vulnerable populations to be assessed and for interventions to be specified in response to climate-related events [36]. We suggest that healthcare services should prepare for increased hospitalization rates at least two years post-event for these populations. Increasing preventive activities in community healthcare settings offers a potential path for mitigating the expected long-term health impacts of wildfires, especially among low-SES populations and those who suffer from poor health. Combining these insights when planning future health services can help communities increase their resilience to wildfires and other climate-related extreme events and prevent likely future health burdens.

## Figures and Tables

**Table 1:** Socio-demographic characteristics of the study population.

Variable		Study Population (*n* = 106,595)	
		*n*	%
Gender	Female	55,291	51.8
	Male	51,304	48.1
Country of birth	Israel	76,735	72.0
	Other	29,860	28.0
Median age at exposure		37 (IQR = 17–56)	
Mean SES at exposure		6.74 (*SD* = 2.01, rank 2–10)	
Registry	Cardiovascular	7925	7.4
	Overweight	36,850	34.6
	Obstructive pulmonary disease	1253	1.2
	Diabetes	27,425	25.7

**Table 2:** Socio-demographic characteristics of study participants in different registries.

Disease	Variable		
Cardiovascular *n* = 7925 (7.4%)	Median age		69 (IQR = 57–78)
	SES		6.47 (*SD* = 2.18, 2–10)
	Gender	Male	4706 (59.4%)
	Birth country	Israel	3856 (48.7%)
Obstructive pulmonary disease *n* = 1253 (1.2%)	Median age		70 (IQR = 64–77)
	SES		6.19 (*SD* = 2.21, 2–10)
	Gender	Male	596 (47.6%)
	Birth country	Israel	556 (44.4%)
Diabetes *n* = 27,425 (25.7%)	Median age		61 (IQR = 50–79)
	SES		6.39 (*SD* = 2.53, 2–10)
	Gender	Male	12,605 (46%)
	Birth country	Israel	13,623 (49.7%%)
Overweight *n* = 36,850 (34.6%)	Median age		50 (IQR = 36–62)
	SES		6.51 (*SD* = 2.11, 2–10)
	Gender	Male	18,789 (51%)
	Birth country	Israel	22,801 (61.9)

**Table 3:** Results of the final regression model.

Variables		B	Exp(B)		95% Confidence Interval for Exp(B)	
				Sig.	Lower	Upper
Gender	Male	0.081	1.085	<0.001	1.064	1.106
	Female		1			
	Age at exposure (years)	0.028	1.028	<0.001	1.028	1.029
Study periods	2nd year post exposure	0.240	1.272	<0.001	1.154	1.401
	1st year post-exposure	0.220	1.246	<0.001	1.130	1.372
	One year pre-exposure		1			
Registry	With underlying morbidity	0.465	1.593	<0.001	1.466	1.730
	With no underlying morbidity		1			
SES categories	High	−0.388	0.678	<0.001	0.621	0.741
	Medium	−0.066	0.936	0.141	0.857	1.022
	Low		1			
Interactions						
	With morbidity × High SES × 2nd year post-exposure	−0.138	0.871	0.121	0.732	1.037
	With morbidity × High SES × 1st year post-exposure	−0.156	0.856	0.080	0.719	1.019
	With morbidity × High SES × 1 year pre-exposure	−0.069	0.933	0.188	0.842	1.034
	With morbidity × Medium SES × 2nd year post-exposure	−0.190	0.827	0.032	0.696	0.983
	With morbidity × Medium SES × 1st year post-exposure	−0.218	0.804	0.014	0.676	0.957
	With morbidity × Medium SES × 1 year pre-exposure	−0.106	0.899	0.041	0.812	0.996
	With morbidity × Low SES × 2ndyear post-exposure	−0.074	0.928	0.186	0.831	1.037
	With morbidity × Low SES × 1st year post-exposure	−0.119	0.888	0.036	0.795	0.992
	With morbidity × Low SES × 1 year pre-exposure		1			
	With no morbidity × High SES × 2nd year post-exposure	−0.167	0.847	0.006	0.751	0.954
	With no morbidity × High SES × 1st year post-exposure	−0.091	0.913	0.138	0.810	1.030
	With no morbidity × High SES × 1 year pre-exposure		1			
	With no morbidity × Medium SES × 2nd year post-exposure	−0.220	0.802	<0.001	0.711	0.905
	With no morbidity × Medium SES × 1st year post-exposure	−0.160	0.852	0.009	0.755	0.961
	With no morbidity × Medium SES × 1 year pre-exposure		1			

## Data Availability

The data that support the findings of this study are available from Maccabi Health Services (MHS), but restrictions apply to the availability of these data, which were used under license for the current study and so are not publicly available. Data are, however, available from the authors upon reasonable request and with the permission of MHS.

## Appendix A

**Table A1:** Estimated means of hospitalization rates during study periods based on a Poisson regression model.

Socio-Economic Status	Period	Hospitalization Rate		
		Mean	95% Wald Confidence Interval	
			Lower	Upper
Participants with underlying morbidity (*n* = 49,629)				
High	One year pre-exposure	0.11	0.11	0.11
	One year post-exposure	0.13	0.12	0.13
	2nd year post-exposure	0.13	0.13	0.14
Medium	One year pre-exposure	0.15	0.14	0.15
	One year post-exposure	0.16	0.16	0.17
	2nd year post-exposure	0.17	0.17	0.18
Low	One year pre-exposure	0.17	0.17	0.18
	One year post-exposure	0.19	0.19	0.20
	2nd year post-exposure	0.21	0.20	0.21
Participants with no background illnesses (*n* = 56,966)				
High	One year pre-exposure	0.07	0.07	0.08
	One year post-exposure	0.08	0.08	0.09
	2nd year post-exposure	0.08	0.08	0.08
Medium	One year pre-exposure	0.10	0.10	0.11
	One year post-exposure	0.11	0.10	0.11
	2nd year post-exposure	0.10	0.10	0.11
Low	One year pre-exposure	0.11	0.10	0.12
	One year post-exposure	0.14	0.13	0.15
	2nd year post-exposure	0.14	0.13	0.15
